# Gut microbiota profiles in pediatric atopic dermatitis and their relationship with skin microbiota: an Indonesian case-control study

**DOI:** 10.3389/fmicb.2026.1864438

**Published:** 2026-07-13

**Authors:** Raden Mohamad Rendy Ariezal Effendi, Hok Bing Thio, Robert Kraaij, Chrysanti Murad, Reiva Farah Dwiyana, Oki Suwarsa, Tamar E. C. Nijsten, Luba M. Pardo

**Affiliations:** 1 Department of Dermatology, Erasmus MC, University Medical Center Rotterdam, Rotterdam, Netherlands; 2 Department of Dermatology, Venereology, and Aesthetics, Faculty of Medicine Universitas Padjadjaran, Dr. Hasan Sadikin Hospital, Bandung, West Java, Indonesia; 3 Department of Internal Medicine, Erasmus MC, University Medical Center Rotterdam, Rotterdam, Netherlands; 4 Division of Microbiology, Department of Biomedical Sciences, Faculty of Medicine Universitas Padjadjaran, Bandung, West Java, Indonesia

**Keywords:** 16S rRNA sequencing, dermatitis and atopic, gut microbiota, Indonesia, pediatric, predicted functional pathways, skin microbiota, skin-gut correlations

## Abstract

**Introduction:**

The bacterial gut microbiota has been implicated in the pathophysiology of atopic dermatitis (AD), yet most studies have focused on Western populations and have typically examined the gut or skin microbiota in isolation. Evidence for interactions between skin and gut microbiota in inflammatory skin diseases remains limited, particularly in pediatric cohorts from non-Western settings. Here, we profiled the bacterial gut microbiota in children with AD from Indonesia, and evaluated the relationship with bacterial skin microbiota using paired gut and skin samples from the same individuals.

**Methods:**

Skin and fecal swabs were collected from children and adolescents aged 4–18 years (111 AD cases and 107 controls) recruited from the Pediatric Dermatology Clinic of Dr. Hasan Sadikin General Hospital, an urban tertiary-care referral center in Bandung, West Java, Indonesia. Both skin and gut bacterial microbiota was profiled using 16S rRNA sequencing and bacteria were identified using DADA2 pipeline, previously published skin microbiota data from the same participants were paired with the gut microbiota data for skin-gut analyses. Alpha diversity was compared between AD cases and controls, beta diversity assessed using permutational multivariate analysis of variance (PERMANOVA) and univariable differential analysis was tested using ANCOM-BC2. *In silico* functional pathway analyses was performed with PICRUSt2. Correlation analysis between the bacterial gut microbiota and the skin microbiota was performed using SparXCC.

**Results:**

Gut alpha diversity did not differ between AD cases and controls, although hand feeding was associated with a higher Shannon diversity (β = 0.20; adjusted *p* = 0.041). Gut microbiota composition was associated with birth method, maternal education, and age, while AD status was not significant. We found differences in the composition of between *Dialister invisus*, *Parabacteroides*, *Lachnospiraceae* UCG-009, and *Flavonifractor* in AD. Furthermore, we identified 24 differentially abundant predicted pathways. CMP-8-amino-3,8-dideoxy-D-manno-octulosonate biosynthesis was more abundant in AD, while palmitoyl ethanolamide biosynthesis was more abundant in control. Correlation analysis showed modest to weak correlations between skin and gut microbiota. Fourteen skin–gut pairs showed different correlation between AD and controls. ASV70 (*Staphylococcus capitis/caprae*)–*Butyrivibrio* pair was identified as an AD-enriched associations, whereas ASV173 (*Staphylococcus nepalensis*)–*Odoribacter* was identified as control-enriched association.

**Conclusion:**

Gut microbiota differences by AD were modest. ASV-level, predicted stool functional pathways, and skin–gut correlation analyses suggested selective cross-site associations.

## 1 Introduction

Atopic dermatitis (AD) is a chronic, relapsing inflammatory skin disease characterized by erythematous, hyperpigmented or lichenified skin lesions with an age-specific anatomic distribution (Weidinger et al., 2018; Carr et al., 2024). It is typically accompanied by intense pruritus and a substantial impairment on quality of life (Weidinger et al., 2018). AD is a complex multifactorial condition where genetic susceptibility, exposome, and alterations in the human microbiome contribute to disease development and severity (Stefanovic et al., 2020). Importantly, AD is not only a skin-limited disorder but also part of the broader spectrum of atopic diseases, including food allergy, asthma, and allergic rhinitis (Ständer, 2021).

The advances in next-generation sequencing techniques has expanded the evidence showing that the skin microbiome plays a critical role in AD, with several studies highlighting the involvement of *Staphylococcus aureus* colonization (Geoghegan et al., 2018; Edslev et al., 2021). There is also growing evidence that the gut microbiota is also involved in AD pathophysiology. The role of the gut microbiome in the pathophysiology of AD can be explained from different angles, including alterations in immunological regulation (Lee et al., 2018), dietary variation (Wastyk et al., 2021), and treatment response to AD systemic therapy, among others (Fang et al., 2021). The term skin-gut axis term has recently been coined to describe the tight interactions between the skin and gut microbiome, not only for AD but also for other chronic inflammatory skin conditions (De Pessemier et al., 2021; Hou et al., 2025). In microbiome studies, alpha diversity refers to microbial diversity within a sample, including the number of taxa present and their relative distribution, whereas beta diversity describes differences in microbial community composition between samples or groups (Goodrich et al., 2014). In some studies, lower alpha diversity in the bacterial gut microbiota has been observed in AD cases when compared with sex- and age-matched controls (Ye et al., 2021), with *Bifidobacterium* and *Bacteroides* being lower in AD (Kim and Kim, 2019). In addition, other studies have highlighted the role of *Akkermansia*, *Bifidobacterium*, and *Faecalibacterium* bacteria in immune-related diseases, including AD (Climent et al., 2021; Effendi et al., 2022).

Despite the growing literature on the influence of the skin-gut axis in the development and severity of AD, we still know little about how these systems interact, because most studies examine the microbiome of the skin and gut separately, and only a very limited number of studies have integrated both anatomical locations within the same participants, including individuals with AD and non-atopic controls (Łoś-Rycharska et al., 2021; Zhang et al., 2024). Analyzing skin–gut composition correlations in cases and controls can highlight microbes that tend to co-occur together across skin and gut and can provide support for identifying which specific bacteria are more likely to interact with one another (De Pessemier et al., 2021; Rios-Carlos et al., 2024; Hou et al., 2025).

Furthermore, most studies on the skin and gut microbiome in AD have been conducted in high-income Western cohorts, limiting generalizability to other regions with different cultural and genetic and environmental backgrounds (Tian et al., 2023). Indonesia, one of the world’s most populous countries, has experienced an increasing prevalence of pediatric AD in recent decades (Tsai et al., 2019; Cheng et al., 2021; GBD 2021 Asia Allergic Disorders Collaborators, 2025). Indonesia has distinct cultural and lifestyle practices (e.g., had feeding habits and local dietary patterns), which are likely to contribute to microbiota composition (Khine et al., 2020; Effendi et al., 2026). Despite this, microbiome studies in Indonesian children remain scarce, despite the fact that AD is a substantial pediatric health concern in Indonesia (Diana et al., 2014). In this study, we aimed to characterize the bacterial gut microbiota in children with AD sampled from a tertiary-care referral hospital in Bandung, West Java, Indonesia, and compared the profiles with those of matched controls. Furthermore, we estimated the correlations between the bacterial gut composition of this dataset and data from the bacterial skin microbiota from the same case-control dataset that has been recently described (Effendi et al., 2026).

## 2 Materials and methods

### 2.1 Study population and design

This was case-control study approved by the Health Research Ethics Committee of Dr. Hasan Sadikin General Hospital, Bandung, West Java, Indonesia (reference: LB.02.01/X.6.5/111/2022). The study was carried out in accordance with the ethical guidelines of the World Medical Association Declaration of Helsinki. Written informed consent was obtained from parents or legal guardians before inclusion.

The study included Indonesian children and adolescents aged 4–18 years from April to December 2022, divided into two groups: children with AD and controls. Participants were recruited from the Pediatric Dermatology Clinic at Dr. Hasan Sadikin General Hospital. Full-body skin examination was performed and AD diagnosis were confirmed by Pediatric Dermatologists (R.M.R.A.E and R.F.D) according to the Hanifin and Rajka Criteria (HRC) (Akan et al., 2020). Disease severity was assessed using the Scoring Atopic Dermatitis (SCORAD) Index, which categorized participants into three severity groups: mild AD (SCORAD < 25), moderate AD (SCORAD 25–50), and severe AD (SCORAD > 50) (Rullo et al., 2008; Barbarot et al., 2024). AD group included children with a previous history of AD and prior AD-related treatment, as well as children who were newly diagnosed with AD during recruitment. To minimize the potential influence of recent treatment exposure on microbiota composition, participants in both the AD group and control were excluded if they had used systemic antibiotics, corticosteroids, or probiotic within the past 2 months. For the AD group, additional restrictions included no topical corticosteroid or topical antibiotic use within the previous 7 days. Participants were instructed to avoid using emollients for 24 h and to avoid showering for at least 6 h before sampling.

The control group included children without atopic or inflammatory dermatological conditions. Controls were recruited from siblings or relatives accompanying children with AD to the pediatric dermatology clinic, as well as from children living in the same neighborhoods as the AD cases or in other urban areas within Bandung. These controls were invited to participate through local outreach methods such as posters and flyers. Additionally, non-atopic children attending the pediatric dermatology clinic for other reasons were included. Participants with any history of atopic conditions (e.g., AD, asthma, allergic rhinitis, food allergies), inflammatory or infectious skin disorders, and acute intestinal symptoms were excluded from the study. Controls were selected to match the age and sex distributions of the AD cases. Whenever possible, non-atopic siblings of the AD cases were included as non-matched controls to ensure similar socio-environmental exposures that could influence the skin and gut microbiota. A total of 20 sibling-controls were included in the control group.

Comprehensive demographic data, including variables relevant to microbiota composition were collected through structured interviews with parents or guardians, followed by skin microbiome sampling. Family income was self-reported and categorized using thresholds adjusted for Bandung, based on data from the Indonesian Central Bureau of Statistics (Statistik, 2023). All assessments took place at the Pediatric Dermatology Clinic of Dr. Hasan Sadikin General Hospital in Bandung, West Java, Indonesia.

### 2.2 Microbial sample collection

#### 2.2.1 Skin samples

Skin microbiota sampling has been described in detail previously (Effendi et al., 2026). In short, skin swabs were collected after clinical assessment using flocked swabs with prefilled preservative tube kits (Copan ^®^ 608CS01R; FLOQSwab ^®^ with eNAT^®^ 1.0 mL). For the present paired skin–gut analyses, we used lesional skin samples from AD cases and skin samples from controls. Lesional samples were obtained from active lesions, preferably from the antecubital or popliteal folds; when no active lesions were present in these areas, samples were taken from the most prominent affected site. Skin samples from controls were collected from the dorsal distal forearm. Each 5 × 5 cm skin area was swabbed for 60 s with firm pressure using a pre-moistened swab, first horizontally across the surface with the moistened side and then vertically with the dry side. Samples were stored at −80°C until DNA extraction. Skin and fecal specimens were linked using a participant-specific unique identifier to ensure correct matching for paired skin–gut analyses.

#### 2.2.2 Fecal samples

Fecal samples were used as a non-invasive proxy for gut microbiota (Tang et al., 2020). Participants self-collected a fecal specimen at home using flocked swabs with prefilled preservative tubes (Copan FLOQSwab^®^ with eNAT^®^ 1.0 mL). Caregivers were instructed that the child should urinate beforehand and to use the provided feces-catcher to prevent urine contamination. The collection kit included detailed, step-by-step instructions and a checklist to document the date and time of defecation and sampling. After collection, tubes were kept at room temperature in accordance with the kit guidance until retrieval by research staff, transported in a cool box filled with ice packs to the Biomedical Sciences Laboratory (Faculty of Medicine, Universitas Padjadjaran), and stored immediately at −80°C.

### 2.3 DNA extraction and 16S rRNA gene polymerase chain reaction amplification and sequencing

Fecal DNA was extracted using the QIAamp PowerFecal Pro DNA Kit (Qiagen), following the manufacturer’s protocol. Samples (up to 250 mg) were mixed with Solution CD1, vortexed, and homogenized. After centrifugation, the supernatant was treated with Solution CD2, then centrifuged again, and up to 700 μL of the supernatant was transferred. Solution CD3 was added, and the lysate was loaded onto an MB Spin Column. After centrifugation, the column was washed with Solutions EA and C5. DNA was eluted with Solution C6 and stored at −80°C until analysis.

In accordance with the material transfer agreement (HK.01.07/H/2918/2023), DNA samples were transported on dry ice to Rotterdam, The Netherlands via World Courier, with temperature monitoring. For fecal DNA sequencing, the V3–V4 regions were amplified. Fecal samples were sequenced on the Illumina NextSeq2000 platform at the Genomics Core Facility of Erasmus University Medical Center, Rotterdam, the Netherlands (Erasmus MC). Positive controls were processed alongside study samples to monitor library preparation, sequencing, and taxonomic assignment, including a commercial microbial reference standard and an internal reference standard provided by the Genomics Core Facility (Zymo Research, Irvine, CA, United States; internal communication).

### 2.4 Analysis

#### 2.4.1 Bioinformatic analysis

Raw sequencing reads from the Illumina NextSeq2000 platform were processed into sample-specific FASTQ files using custom scripts in QIIME1 (version 1.9.1) (Caporaso et al., 2010). Primer sequences were removed using TagCleaner (v0.16) (Schmieder et al., 2010). The DADA2 pipeline (v1.16) in R (version 4.0.0) was applied to process and filter the sequences, enabling the identification of amplicon sequence variants (ASVs), which allowed for more accurate error correction without the need for clustering at predefined similarity thresholds (Callahan et al., 2016). After denoising, chimeric sequences were removed using DADA2, and the resulting dataset of chimera-free ASVs was used for downstream analyses.

ASVs represent unique biological sequences directly derived from the raw reads, and offer higher resolution and reproducibility compared to traditional operational taxonomic units (OTUs) (Callahan et al., 2016; Callahan et al., 2017). Taxonomic classification was carried out using the RDP naïve Bayesian classifier within DADA2 (Wang et al., 2007), with the SILVA SSU rRNA reference database (release 138.1) as the reference (Quast et al., 2012). ASV counts and their corresponding taxonomic information were then compiled into a phyloseq object to generate abundance and taxonomy tables (McMurdie and Holmes, 2013). Analyses were performed at the ASV level, with community composition visualized as relative-abundance bar plots at the phylum and genus levels.

#### 2.4.2 Bacterial microbiota filtering and pairing of samples

Quality control of the gut microbiota was performed using the prevalence function from the “Decontam” R package (Davis et al., 2018). This function compares the prevalence of ASVs in true samples and negative controls to estimate the likelihood that an ASV is a contaminant. For stool samples, a *P* = 0.20 threshold was applied to identify potential contaminants based on the histogram (Supplementary Figure 1). The minimum read depth was set at 50,000 reads, based on the plateau of the rarefaction curve (Supplementary Figure 2). Gut ASVs were further filtered using a 10% prevalence threshold. The filtering steps are summarized in the flow diagram presented in Supplementary Figure 3.

#### 2.4.3 Data analysis

Demographic and clinical characteristics were summarized using proportions for categorical variables and mean (SD) or median (IQR) for continuous variables, as appropriate. Differences between AD cases and controls were evaluated using Chi-squared tests for categorical variables and independent *t*-tests or Mann–Whitney U tests for normally and non-normally distributed continuous variables, respectively.

##### 2.4.3.1 Profile of bacterial gut microbiota

We first estimated alpha diversity (Chao1 and Shannon) in AD cases and controls. Differences in these parameters between cases and controls were tested using a multivariable linear regression adjusting for age, body mass index (BMI) categories, parental education (categorized as intermediate for secondary school and vocational training, and high for university degrees or higher), family income (high or very high), birth method (cesarean section or vaginal delivery), breastfeeding status (exclusive or non-exclusive), feeding habits (hand-fed or spoon-fed), frequency of fiber intake (low or high), frequency of sugar intake (low or high), parental history of atopy (yes or no), and DNA concentration.

Beta diversity was assessed using Bray–Curtis distance and visualized with principal coordinate analysis (PCoA) (Lozupone and Knight, 2005). Prior to distance calculation, zero counts were imputed using multiplicative replacement, and data were Hellinger-transformed (Palarea-Albaladejo and Martín-Fernández, 2015; Chen et al., 2021). The association between individual variables and their contributions to the overall variation of the microbiota composition were tested using PERMANOVA (function “adonis2,” R package “vegan,” version 2.6.4 with Bray-Curtis distances) (Kelly et al., 2015), using the same variables as in the alpha diversity models.

Univariate differential abundance between AD cases and controls was evaluated using Analysis of Compositions of Microbiomes with Bias Correction (ANCOM-BC2, package “ancombc” version 2.12.0) (Lin and Peddada, 2024). Models included the same variables as above, and multiple testing correction was performed using the Benjamini-Hochberg method (Benjamini and Hochberg, 1995), and statistical significance was defined as an adjusted *p* < 0.05. To minimize noise from rare taxa, only taxa present in at least 10% of samples were included in the ANCOM-BC2 analysis.

##### 2.4.3.2 *In silico* functional pathway annotation

To predict the metabolic pathways associated with the stool microbiota, we used PICRUSt2 (phylogenetic investigation of communities by reconstruction of unobserved states). PICRUSt2 is a computational approach that predicts the functional abundance of a metagenome using marker gene data and a database of reference genomes (Douglas et al., 2020). We used PICRUSt2v.2.6.3 with parameter defaults. Next, we tested differences in the composition of the identified pathways between AD cases and controls using ANCOMBC2. Pathway names and annotations were assigned according to the MetaCyc database, a reference source of metabolic pathways (Caspi et al., 2019).

##### 2.4.3.3 Correlation between stool and skin bacterial microbiota

We used the filtered data from our recently published study on bacterial skin microbiota and AD from the same case-control dataset (Effendi et al., 2026), to compare skin microbiota composition with gut microbiota composition. For this analysis, we paired skin and gut samples to have the same number of samples for analysis.

We first normalized the data using relative abundances per subgroup (AD skin, skin controls, AD gut, gut controls). Next, we calculated the correlations between skin and gut filtered data per group, namely: cases and controls. For this, we used SparXCC (Sparse Cross-Correlations between Compositional data) to quantify cross-correlations between skin and gut abundances, with parameters pseudocount = 1 and iterations = 10. This method considers the sparsity and compositionality of microbiota (Jensen et al., 2024). After calculation of the correlation matrices per group, we calculated the differences in correlations between AD and controls using the following steps:

(1) We used Fisher’s z-transformation (atanh), to linearize the correlations
z = tanh-1(r); where r is the correlation matrix
(2) We calculated the standard error:
SE = sqrt (1/(Nca −3) + 1/(Ncon −3);with Nca the number of AD cases and Ncon the number of controls
(3) We computed the difference in the statistics:
zij = SEzij (cases) –zij (ctrl)
(4) We computed the *p*-value
p_ij = 2 * [1–Φ(| z_ij|)]
with Φ representing the cumulative distribution function (CDF) of the standard normal distribution:
Φ(x) = P (Z ≤ x), where Z ∼ N (0, 1)

The differences in the correlations were graphically represented in a bubble correlation analysis.

##### 2.4.3.4 Sensitivity analysis

**FIGURE 1:**
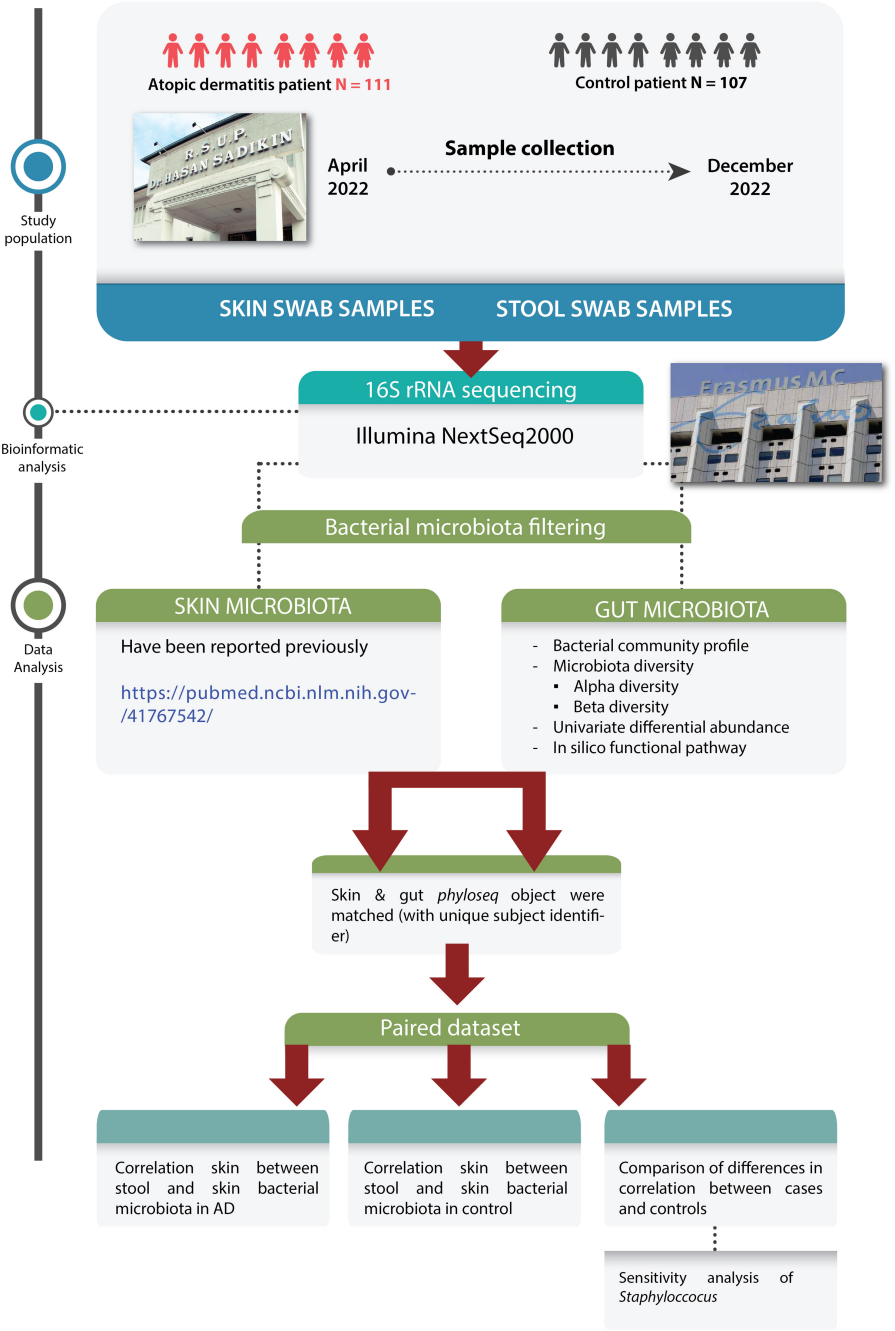
Flow diagram summarizing the study design and analytical workflow. After bacterial microbiota filtering, skin microbiota analyses had been reported previously (Effendi et al., 2026), whereas gut microbiota analyses in the current study included bacterial community profiling, alpha diversity, beta diversity, univariate differential abundance, and *in silico* functional pathway analysis. Skin and gut phyloseq objects were then matched using a unique subject identifier to generate a paired dataset for downstream skin–gut correlation analyses in AD and controls, comparison of differential correlations between groups, and sensitivity analysis focused on *Staphylococcus.*

We also focused on ASV belonging to the genus *Staphylococcus* given the relevance of species within this genus in AD, and repeated the correlation analysis as above. Because not all relevant *Staphylococcus* ASVs could be assigned to the species level using the DADA2 pipeline, targeted phylogenetic analysis was used to support taxonomic interpretation. The methodological details of this analysis have been described previously (Effendi et al., 2026). *Staphylococcus* ASVs assigned to species level based on this phylogenetic analysis are indicated by the term “likely” in the Results. Figure 1 summarizes the analytical workflow. All analyses were conducted using R software, version 4.5.2 (R Core Team, 2023).

## 3 Results

A total of 218 participants were included, 111 AD cases and 107 controls (Effendi et al., 2026). Four participants did not return their fecal samples, resulting in 214 fecal samples. Since skin microbiota data were available for these same participants, paired skin-gut microbiota data were included for 214 participants in the subsequent skin-gut analysis. The AD group was nearly 2 years younger than the control group (mean age 8.35 ± 3.51 vs. 9.91 ± 3.79 years, *P* = 0.002). The demographics of the study participants are summarized in Table 1.

**TABLE 1:** Participants.

Demographics	AD (*n* = 111)	Controls (*n* = 107)	*P*-value
Age (years) mean ± SD	8.35 ± 3.51	9.91 ± 3.79	0.002*
Age (years) median (IQR)	8.00 (6.0)	10 (6.0)	0.003*
Gender			0.95
Male	52 (46.8%)	52 (48.6%)	
Female	59 (53.2%)	55 (51.4%)	
BMI			0.07
Normal	77 (69.4%)	87 (81.3%)	
Overweight	34 (30.6%)	20 (18.7%)	

Statistical significance for differences between groups is marked with * for *p* < 0.05.

### 3.1 Bacterial community profiles

The gut microbiota composition showed broadly similar relative abundance profiles between AD cases and controls at both the phylum and genus levels (Supplementary Figure 4). *Bacteroidota* and *Firmicutes* were predominant in both groups at the phylum level (Supplementary Figure 4A). At the genus level, the most abundant genera in both groups included *Prevotella_9, Coprococcus, Faecalibacterium*, and *Dialister*, with no major visual differences between AD cases and controls (Supplementary Figure 4B). Likewise, stratification by AD severity did not reveal a clear severity-dependent pattern in the dominant genera (Supplementary Figure 4C).

### 3.2 Microbiota diversity

Gut alpha diversity (Shannon diversity and Chao1) was not significantly different in AD cases compared to controls (Supplementary Figure 5) and only hand feeding was independently associated with a higher Shannon diversity (β = 0.20, SE = 0.07, adjusted *p* = 0.041; Table 2). In Supplementary Figure 6, the PCoA plot showed substantial overlap between AD cases and controls, with a tendency toward greater dispersion among AD samples. In the adjusted PERMANOVA analyses, gut microbiota composition was most strongly associated with DNA concentration (*R*^2^ = 1.74%, *p* < 0.001), with smaller but significant contributions from birth method, maternal education, and age (Table 3).

**TABLE 2:** Multivariable linear regression of factors with Alpha diversity.

Tested exposure	Chao1 richness			Shannon diversity		
	β	SE	Adjusted *p*	β	SE	Adjusted *p*
Cases (ref:control)	−17.90	13.29	0.35	−0.06	0.08	0.74
Age in years	2.74	1.51	0.17	0.005	0.009	0.78
Father education high (ref:intermediate)	−22.17	17.76	0.35	−0.17	0.111	0.382
Mother education high (ref:intermediate)	−14.75	17.24	0.53	−0.02	0.11	0.89
Family income very high (ref:high)	6.70	12.50	0.67	0.11	0.08	0.38
Birth method section caesarea (ref: vaginal)	−26.75	12.20	0.08	−0.17	0.07	0.137
Exclusive breastfeed (ref: no)	5.11	10.55	0.67	0.005	0.067	0.935
Feeding habit—hand feed (ref:spoon feed)	29.82	11.48	0.0507	0.20	0.07	0.041*
Sugar intake high (ref:low)	4.18	11.007	0.70	−0.07	0.07	0.528
Fiber intake low (ref:high)	−12.89	11.13	0.37	−0.08	0.071	0.453
Atopy mother yes (ref:no)	16.11	12.28	0.35	0.12	0.07	0.382
Atopy father yes (ref:no)	6.75	12.77	0.67	0.03	0.08	0.78
DNA concentration	0.35	0.14	0.07	0.003	0.009	0.787

Multivariable linear regression models assessing associations between tested exposures and alpha diversity metrics (Chao1 richness and Shannon diversity). Beta coefficients (β), standard errors (SE), and adjusted *p*-values are reported. Models were adjusted for relevant demographic, clinical, and technical covariates. Statistically significant associations (adjusted *p*-value) are marked with * for *p* < 0.05.

**TABLE 3:** Unadjusted and adjusted PERMANOVA for gut microbiota composition.

Tested main exposure	Unadjusted *R*^2^ (%)	Unadjusted (*p*-value)	Adjusted *R*^2^ (%)	Adjusted (*P*-value)
Age	1.53	0.001***	0.70	0.049*
Mother education	3.66	0.001***	0.76	0.028*
Father education	2.88	0.001***	0.40	0.634
Family income	2.66	0.001***	0.71	0.052
Atopy mother	0.94	0.015*	0.52	0.246
Atopy father	1.72	0.001***	0.48	0.382
Birth method	1.59	0.001***	0.91	0.009**
DNA concentration	2.19	0.001***	1.74	< 0.001***
ADStatus	2.19	0.001***	0.67	0.064

Multivariable linear regression models assessing associations between tested exposures and alpha diversity metrics (Chao1 richness and Shannon diversity). Beta coefficients (β), standard errors (SE), and adjusted *p*-values are reported. Models were adjusted for relevant demographic, clinical, and technical covariates. Statistically significant associations (adjusted *p*-value) are marked with * for *p* < 0.05, ** for *p* < 0.01, and *** for *p* < 0.001.

### 3.3 Univariate differential abundance with ANCOMBC2

At the genus level, we did not find any bacteria with a significant differential abundance between AD cases and controls. At the ASV level, four ASVs were differentially abundant, all showing higher abundance in AD cases (Supplementary Figure 7). Of these, ASV446 was classified to species level by DADA2 as *Dialister invisus*. The remaining AD-enriched ASVs were assigned to *Parabacteroides* (ASV616), *Lachnospiraceae* UCG-009 (ASV806), and *Flavonifractor* (ASV1538).

### 3.4 Univariate differential abundance of stool functional pathways

**FIGURE 2:**
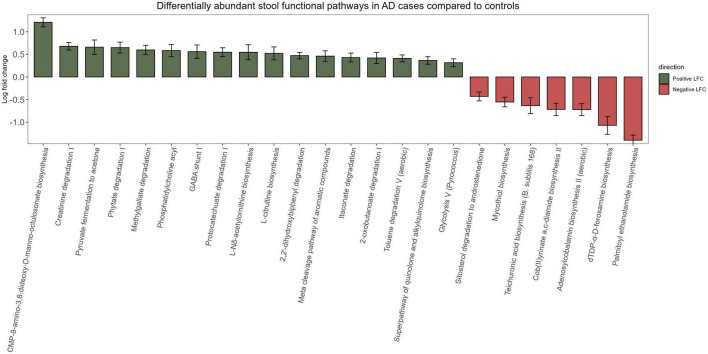
Differentially abundant predicted gut microbial pathways in AD cases compared with controls. Positive log fold change (green bars) indicates higher predicted pathway abundance in AD cases, whereas negative log fold change (red bars) indicates higher predicted pathway abundance in controls.

We identified 24 stool functional pathways that differed significantly in abundance between children with AD and controls (Figure 2). Seventeen pathways were significantly more abundant in AD, whereas seven pathways were more abundant in controls. The pathway with the largest difference in AD was CMP-8-amino-3,8-dideoxy-D-manno-octulosonate biosynthesis (LFC 1.21; SE 0.10). Other pathways more abundant in AD included creatinine degradation I, pyruvate fermentation to acetone, phytate degradation I, methylgallate degradation, phosphatidylcholine acyl, and GABA shunt I, among others. In contrast, pathways that were more abundant in controls were palmitoyl ethanolamide biosynthesis (LFC −1.40, SE 0.11) and dTDP-α-D-forosamine biosynthesis (LFC −1.07, SE 0.20), followed by adenosylcobalamin biosynthesis II (aerobic) and cob(II)yrinate a,c-diamide biosynthesis II, among others.

### 3.5 Correlation between stool and skin bacterial microbiota

The skin microbiota profiles used for skin-gut analysis, including corresponding skin microbiota results and figures, have been reported previously (Effendi et al., 2026). Lesional AD skin differed from control in both diversity and composition, with a predominance of *Staphylococcus aureus* and *Staphylococcus epidermidis.* Several less commonly reported genera, including *Acetobacter* and *Gluconobacter*, were also more abundant in AD lesional skin. Skin microbial composition was further associated with case-control status, family income, maternal atopy, maternal education, and DNA concentration. Phylogenetic analysis supported clear lineage-level differences among *Staphylococcus* ASVs. These previously reported skin microbiota data were matched with fecal microbiota data from the same participants using the participant-specific identifier and were used for the present cross-site skin–gut correlation analyses.

**FIGURE 3:**
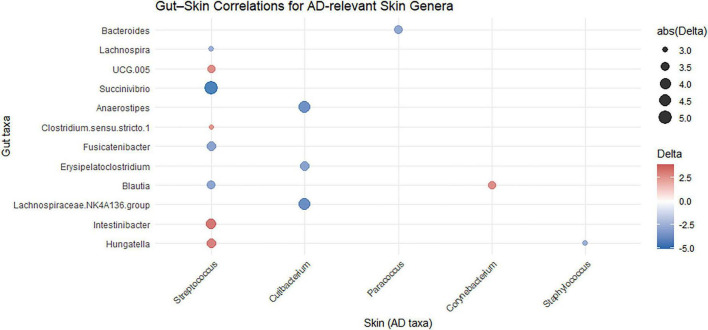
Bubble plot showing significant differential correlations between AD cases and controls for relevant skin and gut genera. Each bubble represents a skin–gut genus pair with a significantly different correlation between groups (adjusted *p* < 0.05). The *x*-axis shows AD-relevant skin genera and the *y*-axis shows gut genera. Bubble color represents the direction of the difference in correlation (delta), where red indicates stronger correlations in AD and blue indicates stronger correlations in controls. Bubble size reflects the magnitude of the difference in correlation (delta).

Genus-level SparXCC analysis, followed by delta-correlation comparison, identified 14 skin–gut genus pairs with statistically significant differences in correlation between AD cases and controls (adjusted *p* < 0.05; Figure 3). Most significant associations involved skin S*treptococcus* (*n* = 8), followed by *Cutibacterium* (*n* = 3), while *Staphylococcus*, *Corynebacterium*, and *Paracoccus* each contributed one association. These pairs included associations between skin *Streptococcus* or *Cutibacterium* and gut genera such as *Succinivibrio, Lachnospira, Lachnospiraceae NK4A136* group, *Blautia, Anaerostipes, Intestinibacter, Hungatella, UCG-005, and Clostridium sensu stricto* 1. Of the 14 significant pairs, five showed stronger correlations in AD cases relative to controls (positive delta), whereas nine showed weaker correlations in AD (negative delta).

#### 3.5.1 Sensitivity analysis

**FIGURE 4:**
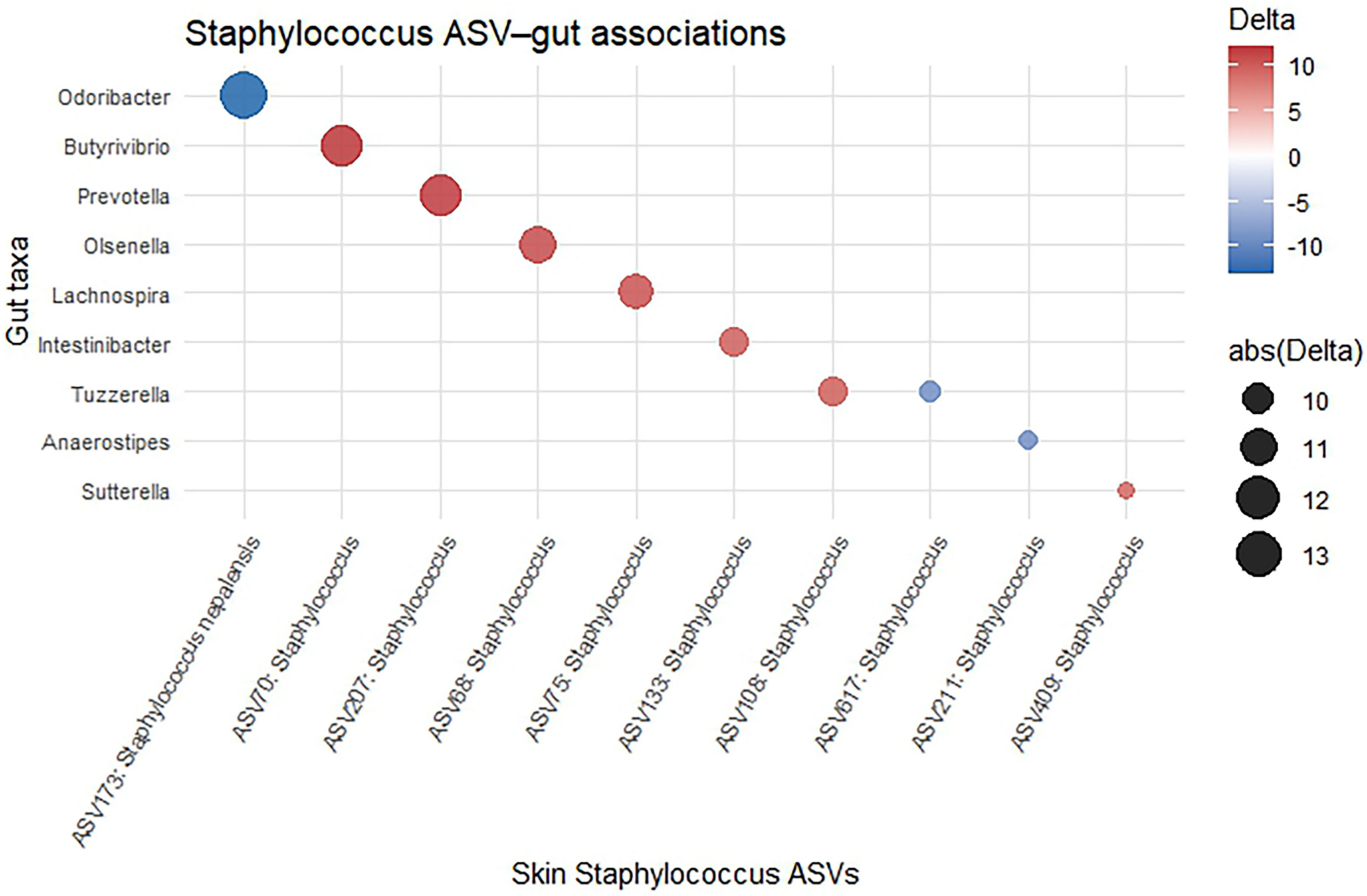
Bubble plot showing significant differential correlations between skin *Staphylococcus* ASVs and gut taxa in children with AD compared with controls. Each bubble represents a skin ASV–gut taxon pair with a significantly different correlation between groups. The *x*-axis shows skin *Staphylococcus* ASVs and the *y*-axis shows gut taxa. Bubble color represents the direction of the difference in correlation (delta), where red indicates stronger correlations in AD and blue indicates stronger correlations in controls. Bubble size reflects the magnitude of the difference in correlation.

**FIGURE 5:**
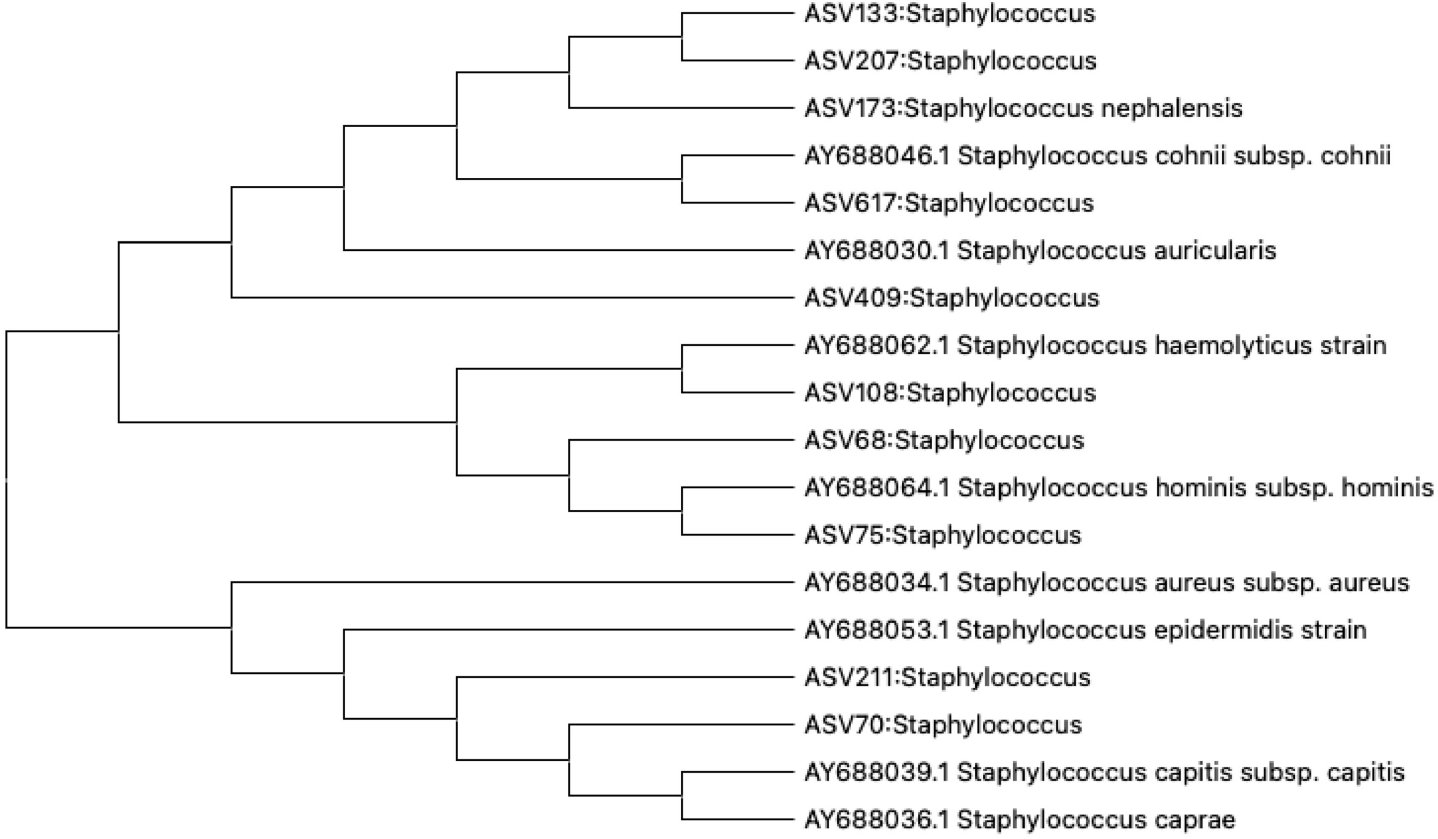
A phylogenetic tree assigned together with reference sequences of *Staphylococcus* species and the significant correlation of *Staphylococcus* ASVs from the differential correlation between AD and controls.

In Figure 4, we show the *Staphylococcus* ASVs–gut associations with the largest differential correlations between AD cases and controls, while the full set of statistically significant associations is provided in Supplementary Figure 8. Among the associations shown in Figure 4, seven were AD-enriched (delta > 0), and three were control-enriched (delta < 0). The strongest case-enriched association was observed for ASV70 (likely *Staphylococcus capitis*/*caprae*) with *Butyrivibrio*, whereas the strongest control-enriched association was observed for ASV173 (*Staphylococcus nepalensis*) with *Odoribacter*. Phylogenetic analysis of *Staphylococcus* ASVs showed clustering with multiple *Staphylococcus* references, supporting species-level interpretation for selected ASVs (Figure 5 and Supplementary Table 1).

## 4 Discussion

In this study, we profiled the bacterial stool microbiota in children with AD and compared it with controls. We found similar alpha and beta diversity metrics in the microbiota between AD and controls and a weak differential composition of ASVs from *Dialister invisus* (ASV446)*, Parabacteroides* (ASV616), *Lachnospiraceae* UCG-009 (ASV806), and *Flavonifractor* (ASV1538) in AD cases when compared with controls. *In silico*-derived stool functional pathway analysis showed an increased abundance of CMP-8-amino-3,8-dideoxy-D-manno-octulosonate biosynthesis in AD, together with pathways involved in fermentation and central carbon metabolism. In contrast, controls were enriched in cobalamin biosynthesis–related pathways. Together with the previously reported skin microbiota findings from the same cohort, these results suggest that microbiota alterations in this population were more pronounced at the skin level than in the gut. Finally, a correlation analysis comparing skin data and gut microbiota in the same dataset showed weak correlations in bacterial abundance between skin and gut in both AD and controls, with most differential correlations being stronger in controls. In the *Staphylococcus* ASV sensitivity analysis, we identified ASV70 (likely *Staphylococcus capitis/caprae*) with *Butyrivibrio* as the strongest cases-enriched association, and ASV173 (*Staphylococcus nepalensis*) with *Odoribacter* as the strongest control-enriched association.

In contrast to other studies, we did not find a significant difference in alpha diversity in the bacterial gut microbiota between cases and controls, which is in line with a recent study also conducted in children with AD (Ismail et al., 2012; Cheung et al., 2023). This contrasts with earlier studies showing a reduced neonatal gut microbial diversity in infants who later developed eczema (Forno et al., 2008). This and results from an earlier study in school children (Hu et al., 2021), showed that overall gut microbial diversity may be more relevant in very early life than in later childhood AD. In addition, since this study is from Indonesian children, it could be argued that this may be a population-specific finding. Evidence of the latter can be illustrated that we found differences in Shannon diversity in relation to hand feeding, suggesting that feeding practices may contribute to variation in gut microbial diversity.

Comparisons of beta diversity did not show a significant overall difference in the microbiome composition between AD and controls. This was in line with the absence of significant differences in gut alpha diversity by AD status. Instead, variation in gut microbiota composition was associated with other variables, particularly with birth method, maternal education, and age. This aligns with evidence that cesarean delivery alters early gut microbial composition, with these differences persisting through infancy and into the first year of life (Rutayisire et al., 2016). While the association between maternal education and gut microbiota in AD is limited, socioeconomic factors, including parental education, are linked to microbiome composition in pediatric population-based studies (Lewis et al., 2021). The association with age reflects the ongoing maturation of gut microbiota, with older children exhibiting more adult-like profiles as microbial composition becomes more complex through childhood and adolescence (Galazzo et al., 2020; Ou et al., 2022).

Although we did not find differential abundance in bacterial composition at the genus level, we identified a higher abundance of *Dialister invisus*, *Parabacteroides*, *Lachnospiraceae* UCG-009, and *Flavonifractor* in AD cases when compared with controls. *Dialister invisus* has been found in oral and gut microbiota studies, the latter in relation to inflammatory bowel disease (Al Radi et al., 2024), and type 1 diabetes (Siljander et al., 2019). In contrast, *Parabacteroides* abundance was directionally consistent with an Italian pediatric cohort reporting higher *Parabacteroides* in AD together with multiple control-associated short chain fatty acid (SCFA)-related genera (Reddel et al., 2019). *Lachnospiraceae* UCG-009 has not been consistently reported in pediatric AD, although other taxa within *Lachnospiraceae* family have been associated with AD; for example, Generation R in Dutch school-age children reported *Lachnospiraceae* associated with lower eczema risk (Hu et al., 2021), whereas a Chinese infant eczema study reported enrichment of *Lachnospiraceae*-related taxa in cases (Zheng et al., 2016). Finally, pediatric AD-specific evidence for *Flavonifractor* remains limited; however, an adult AD gut microbiome studies from China have reported *Flavonifractor* enrichment in cases (Zheng et al., 2016), supporting plausibility and the need for validation in pediatric datasets.

In our study, differential abundance of predicted gut functional pathways indicated differences between AD and controls. These findings are broadly consistent with European-based studies linking pediatric eczema risk to lower SCFAs early in life, supporting a role for altered fermentation ecology in childhood AD (Barman et al., 2024). In AD cases, several pathways mapped to fermentation and central carbon functions, including pyruvate fermentation to acetone and GABA shunt I. While these pathways are not direct butyrate-production pathways, they support the presence of altered fermentation-related metabolism; in particular, the GABA shunt generates succinate, a key cross-feeding metabolite in the gut (Connors et al., 2019; Culp and Goodman, 2023). This is relevant because pediatric AD has been linked to altered SCFA profiles, and SCFAs, particularly butyrate, can strengthen barrier integrity and attenuate AD-like inflammation (Trompette et al., 2022). The strongest AD-associated signal was CMP-8-amino-3,8-dideoxy-D-manno-octulosonate biosynthesis. However, its specific relevance to AD remains unclear and needs further validation. In controls, cobalamin biosynthesis–related pathways including adenosylcobalamin biosynthesis II (aerobic) and cob(II)yrinate a,c-diamide biosynthesis II, were higher, consistent with an adult European metagenomic study reporting reduced vitamin biosynthesis pathways in AD (Díez-Madueño et al., 2025). Palmitoyl ethanolamide biosynthesis was also strongly control-associated. While AD-specific evidence is limited, palmitoyl ethanolamide has been linked to gut barrier and microbiome related effects in experimental and human studies (Couch et al., 2019; Pirozzi et al., 2023). The remaining significant pathways clustered into energy/fermentation (most directly linked to pediatric AD through SCFA evidence), amino-acid/nitrogen metabolism, and dietary/aromatic compound processing. However, because PICRUSt2 provides predicted functional potential rather than measured genes or metabolites, the findings can be considered only explorative (Douglas et al., 2020).

In our paired skin–gut correlation analysis, only a limited number of skin–gut genus pairs showed significantly different correlation between AD cases and controls, and most of these differential correlations were stronger in controls. Several control-enriched pairs involved skin *Cutibacterium* or *Streptococcus* with gut *Anaerostipes*, *Blautia*, and *Lachnospiraceae*-related taxa, which is biologically relevant given their commensal and SCFA-related functions (Chia et al., 2018; Vacca et al., 2020; Liu et al., 2021; Rozas et al., 2021; Yu et al., 2024). Consistent with this, pediatric studies have reported reduced *Blautia* in children with AD, while *Lachnospiraceae*- and *Ruminococcaceae*_UCG-005–related taxa have been associated with a lower risk of eczema (Reddel et al., 2019; Hu et al., 2021). By contrast, fewer AD-enriched differential pairs were observed and were driven mainly by skin *Streptococcus*, including associations with *Hungatella* and *Clostridium sensu stricto 1.* These two gut taxa have previously been linked to eczema risk or early eczema-associated gut dysbiosis in infants (Chan et al., 2021; Cheung et al., 2023). The remaining AD-enriched pairs lacked clear support and should be considered exploratory. Overall, these findings showed weak correlations and suggest a potential disruption of microbial interaction between the skin and gut microbiota, with modest interactions between common skin commensal bacteria and gut bacteria, providing insight into skin-gut axis beyond simple compositional changes.

From an immunological perspective, the gut-skin axis has been proposed as a framework linking gut microbiome alterations, systemic immune regulation, and cutaneous inflammation in chronic inflammatory skin diseases, including AD (De Pessemier et al., 2021; Hou et al., 2025). Gut microbiota alterations may influence systemic immune regulation through microbial metabolites and mucosal immune pathways (Barman et al., 2024), while skin microbiota alterations, particularly *Staphylococcus*-associated dysbiosis, may contribute locally to barrier disruption, keratinocyte activation, and cutaneous inflammation (Geoghegan et al., 2018; Edslev et al., 2021). These processes could theoretically contribute to AD progression by reinforcing systemic and local inflammatory pathways, but they may also act independently rather than representing a single coordinated gut–skin mechanism (Trompette et al., 2022). In our study we could not assess the cause effect of the correlations identified because of the cross-sectional nature of the study. Future longitudinal studies integrating paired skin and gut microbiota with immune, metabolomic, and barrier-function measurements are needed to clarify these interactions.

Given the lack of correlation between skin *Staphylococcus* and other gut bacteria, we performed a sensitivity analysis to try to understand this finding, since we know that the *Staphylococcus* genus plays a major role in AD and was associated with AD in these children (Effendi et al., 2026). We found the slightly differential correlation involved gut taxa with plausible links to microbial metabolite ecology. The strongest control-enriched association paired ASV173 (*Staphylococcus nepalensis*) with *Odoribacter*, whereas the strongest AD-enriched association paired ASV70 (likely *Staphylococcus capitis/caprae*) with *Butyrivibrio*. Prior studies supports the relevance of *Odoribacter* and *Anaerostipes*, as both are linked to SCFA-associated gut ecology, although AD-specific evidence is mixed across studies (Kim et al., 2021; Xue et al., 2023; Snodgrass and Velayudhan, 2026). The strongest case-enriched pair involved *Butyrivibrio*, as a butyrate-associated genus, indicating that differential correlation patterns in AD do not necessarily mirror a simple depletion of all SCFA-related taxa, but may instead reflect reorganization of microbial network structure (Snodgrass and Velayudhan, 2026). *Lachnospira* is also SCFA-related genus but has not been consistently highlighted in pediatric AD (Reddel et al., 2019), whereas *Sutterella*, a mucosa-associated genus, has been reported at higher abundance in pediatric AD, and *Prevotella* shows heterogenous associations (Reddel et al., 2019). Evidence for *Olsenella*, *Intestinibacter*, and *Tuzzerella* in AD remains sparse, and these taxa are best considered exploratory findings rather than reproducible AD-associated markers. To our knowledge, the exact skin *Staphylococcus* ASV**–**gut taxa correlation identified here have not been reported previously in AD, extending current understanding and providing new ASV-level evidence that *Staphylococcus*-related gut–skin correlation in AD is likely species dependent, although further study is needed.

## 5 Strength and limitation

A strength of this study is the inclusion of paired skin and gut microbiota data from a characterized pediatric case–control cohort, allowing cross analyses using comprehensive approaches including diversity, ASV-level differential abundance, predicted stool functional pathways, and skin–gut correlations. It also adds data from an underrepresented population in the AD microbiome literature. However, the cross-sectional design limits causal inference, the single-center urban tertiary setting may reduce generalizability, 16S rRNA sequencing limited species-level resolution, and the predicted functional and correlation findings should be interpreted cautiously.

## 6 Conclusion

In conclusion, in contrast to the skin microbiota alterations previously reported in the same population, gut microbiota differences were more limited, suggesting a less prominent role for the gut microbiota compared with the skin microbiota in pediatric AD. Although selective differences were identified at the *Staphylococcus* ASV level, in predicted functional pathways, and in skin–gut correlation analyses, these findings do not support the presence of a strong, overarching gut–skin microbial axis.

## Data Availability

In line with current Indonesian Ministry of Health regulations on the transfer and use of clinical specimens, and biological materials, information, and their related data, the datasets generated and analyzed in this study cannot be deposited in an open international repository without prior approval and a formal Material Transfer and/or Data Sharing Agreement. The official Ministry of Health Material Transfer Agreement service page, which describes the relevant regulatory basis and request procedure, can be consulted at: https://www.badankebijakan.kemkes.go.id/en/layanan-mta-material-transfer-agreement/. Requests for access to de-identified data for research purposes should be directed to the corresponding author (l.pardocortes@erasmusmc.nl), with the first author/local data access contact copied (rendy.ariezal.effendi@unpad.ac.id), and will be considered in accordance with the required approval and agreement process involving our institutions and the Indonesian Ministry of Health. Data sharing is therefore subject to completion of the required approval and agreements.
