# Supplementary material for: Gut microbiota profiles in pediatric atopic dermatitis and their relationship with skin microbiota: an Indonesian case-control study
Source: Front Microbiol. 2026 Jul 13;17:1864438. doi: 10.3389/fmicb.2026.1864438 (PMC13402473; doi:10.3389/fmicb.2026.1864438)
Supplement: Supplementary file 1 [file Data_Sheet_1.docx]

Supplementary Material

**Figure S1:** Histogram of Gut Decontam Prevalence Threshold **P2**

**Figure S2:** Rarefaction Curve of Gut Sequencing Depth **P3**

**Figure S3:** Flow Diagram Gut Filtering **P4**

**Figure S4:** Relative Abundance of the gut microbiota at the phylum and genus levels, **P5**

stratified by AD severity at the genus level

**Figure S5:** Gut Alpha diversity in AD cases and controls **P6**

**Figure S6:** Gut Beta diversity in AD cases and controls **P7**

**Figure S7:** Gut Differential abundance ASVs between AD and controls **P8**

**Figure S8:** Full set of statistically significant differential correlation between **P9**

*Staphylococcus* ASV-gut in children with AD and controls

**Table S1:** BLAST Results of Key ASVs for Species-Level Identification **P10**

**Figure S1.** Histogram of Gut Decontam Prevalence Threshold


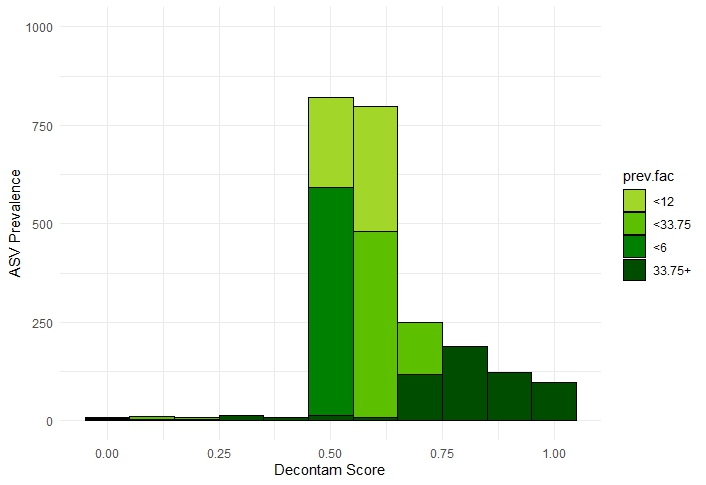


Histogram of ASV Decontam scores calculated using the *prevalence* method from the *Decontam* R package. Bars are colored by prev.fac, a grouping variable based on the number of samples in which each ASV was detected: <6, <12, <33.75, and 33.75+. These brackets were empirically derived from the ASV prevalence distribution (1st quartile = 6, median = 12, 3rd quartile = 33.75) to aid visualization of how detection frequency relates to contaminant classification. A threshold of 0.20 was used to identify potential contaminants.

**Figure S2.** Rarefaction Curve of Sequencing Depth


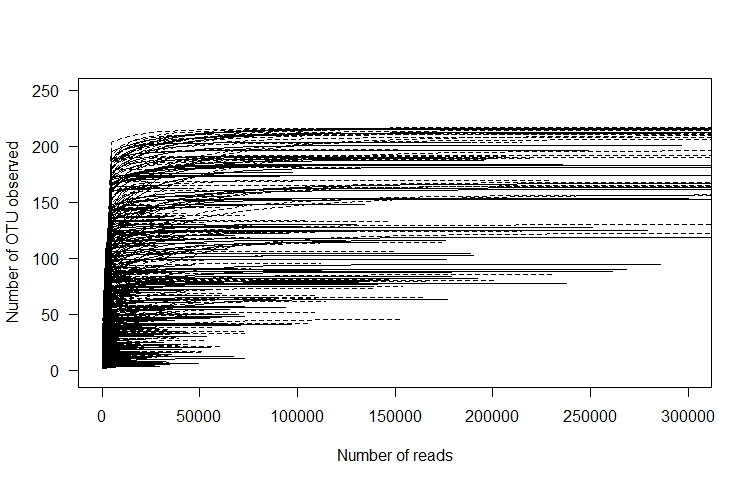
 Rarefaction curves showing the number of observed ASVs as a function of sequencing depth across all samples. Most curves begin to plateau around 50,000 reads, indicating adequate sequencing depth for downstream analysis

.

**Figure S3.** Flow Diagram Gut Filtering

**
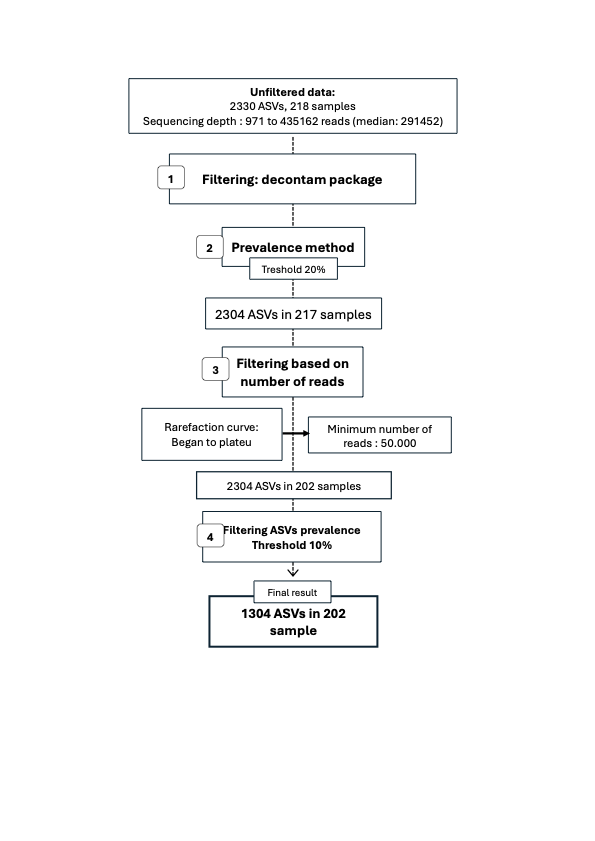
**

Flow diagram summarizing the four main filtering steps applied to the microbiota dataset. ASVs were first filtered by prevalence from *decontam package*, followed by removal of samples with fewer than 50,000 reads, based on rarefaction curve inspection. A 10% ASV prevalence threshold was then applied. The final dataset included 1304 ASVs across 202 samples for downstream analysis.

**Figure S4:** Relative Abundance at phylum, genus level, stratified by AD severity at genus level


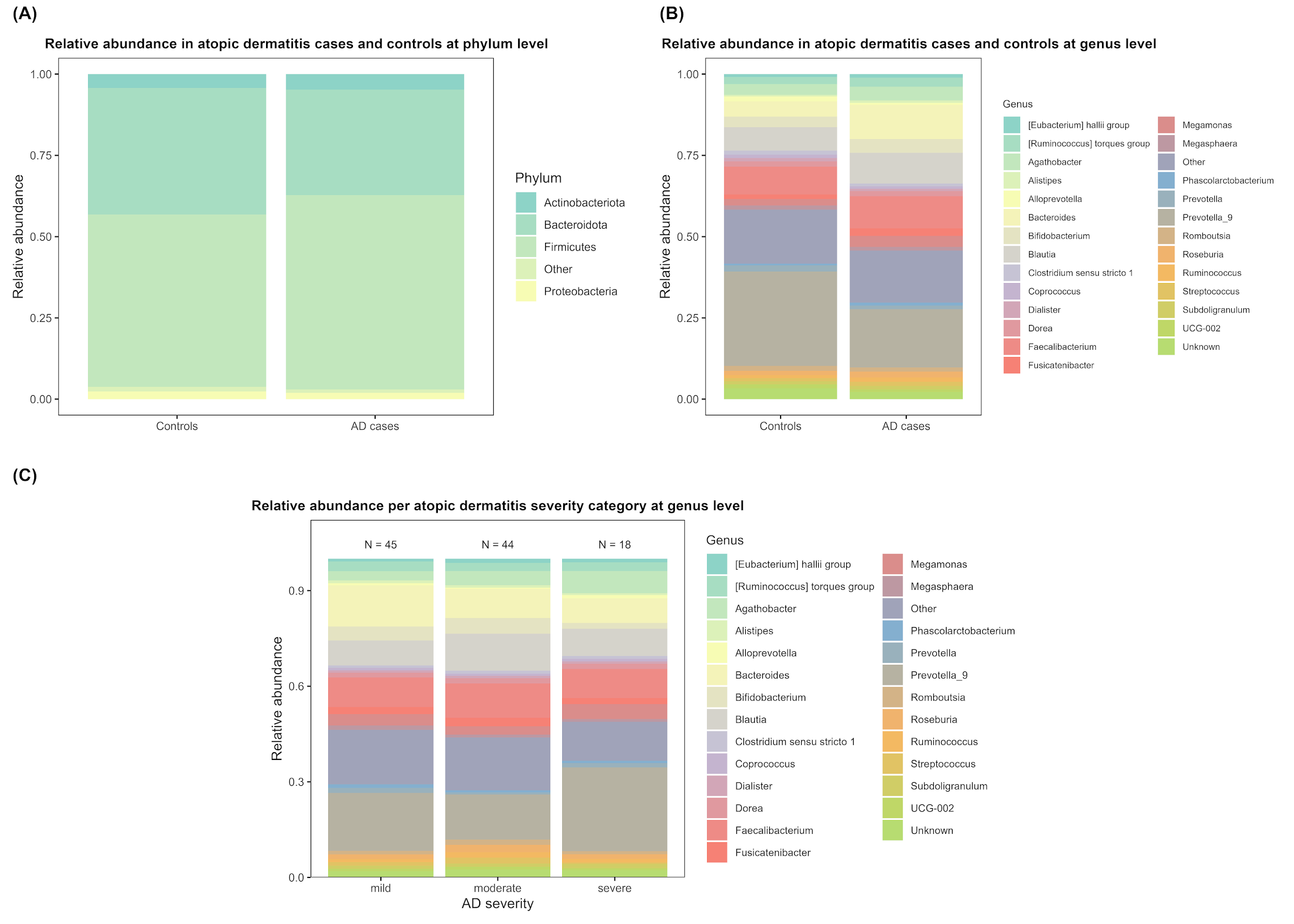


(A) Bar plot showing microbial composition at the phylum level. *Bacteroidota and Firmicutes*predominated in both groups. (B) Genus-level composition most abundant genera in both groups included *Prevotella_9, Coprococcus, Faecalibacterium,*and *Dialister*, with no major visual differences between AD cases and controls. (C) stratification by AD severity did not reveal a clear severity-dependent pattern in the dominant genera.

**Figure S5:** Gut Alpha diversity in AD cases and controls


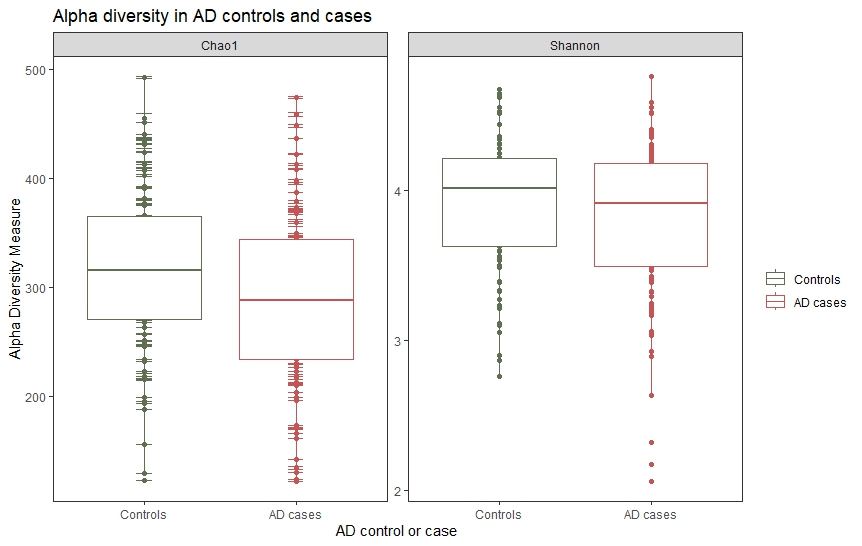


Alpha diversity (Chao1 richness and Shannon diversity) between AD cases and controls. In univariable models, both Chao1 and Shannon index were not significantly different in AD cases compared to controls.

**Figure S6:** Gut beta diversity in AD cases and controls

**
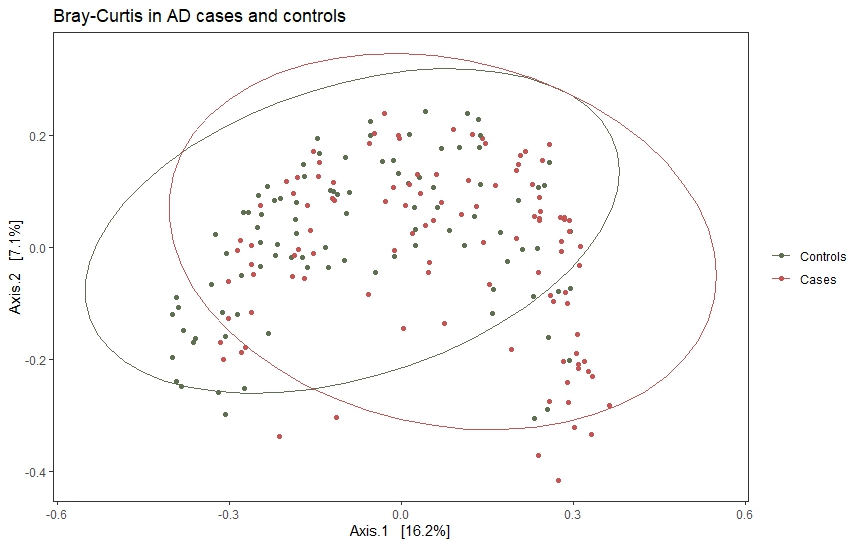
**

Beta diversity in AD cases and controls**.** Bray–Curtis dissimilarities show clearer separation between groups, with greater dispersion among AD cases (Axis 1 = 16.2%, Axis 2 = 7.1%).

**Figure S7:** Gut Differential abundance ASVs between AD and controls

**
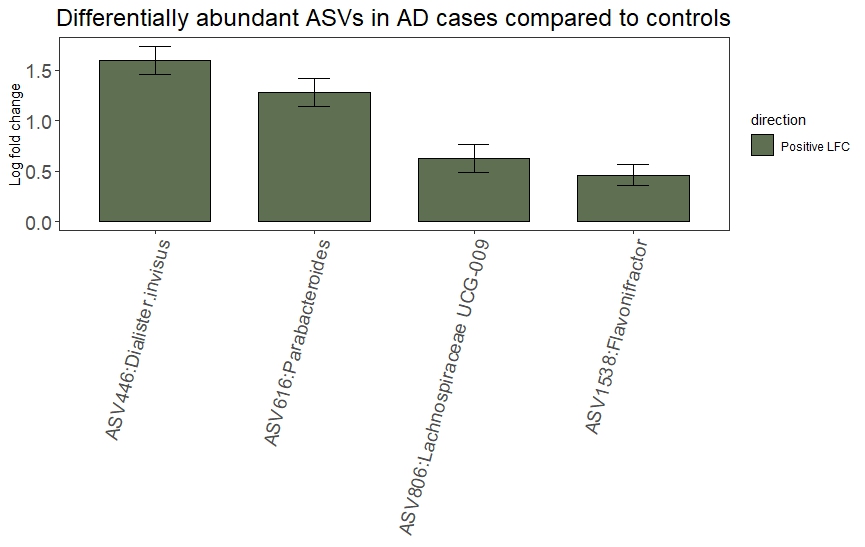
**

Bar plot showing gut amplicon sequence variants (ASVs) identified as differentially abundant between children with AD and controls using multivariable ANCOM-BC2. Positive log fold change indicates higher abundance in AD cases compared with controls. Only ASVs present in ≥10% of samples were included.

**Figure S8:** Full set of statistically significant differential correlation between

*Staphylococcus* ASV-gut in children with AD and controls


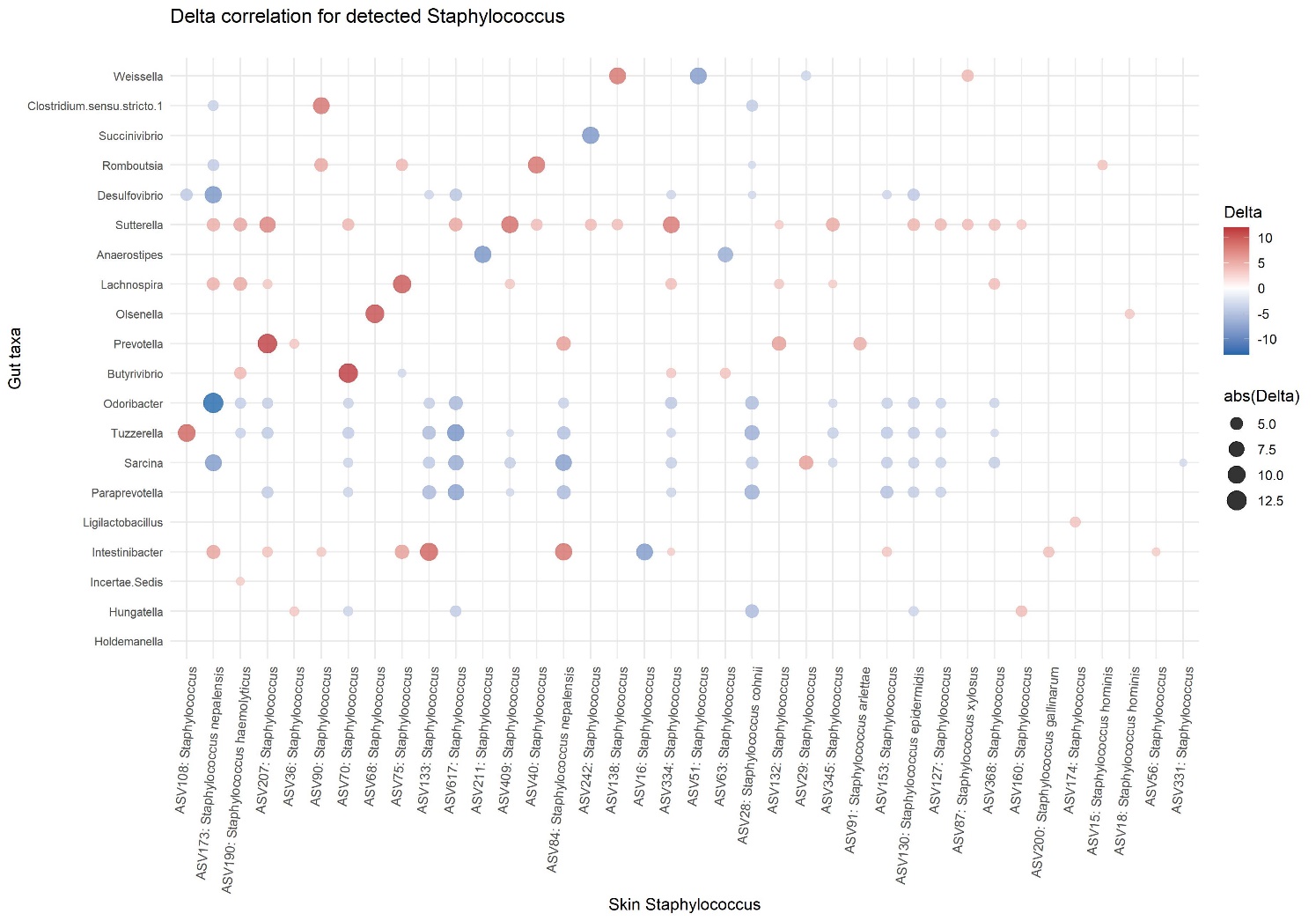


Bubble plot showing the full set of significant differential correlations between skin *Staphylococcus* ASVs and gut taxa in children with AD compared with controls. Each bubble represents a skin ASV–gut taxon pair with a significantly different correlation between groups. The x-axis shows skin *Staphylococcus* ASVs and the y-axis shows gut taxa. Bubble color represents the direction of the difference in correlation (delta), where delta >0 shown stronger correlations in AD and delta<0 indicates stronger correlations in controls. Bubble size reflects the magnitude of the difference in correlation.

**Table S1.** BLAST Results of Key ASVs for Species-Level Identification

Date of search: 26 March 2026

| ASV | Description | Percentage Identical |
| --- | --- | --- |
| ASV68: Staphylococcus | [Staphylococcus hominis strain DM 122 16S ribosomal RNA, partial sequence](https://blast.ncbi.nlm.nih.gov/Blast.cgi#alnHdr_310975092) | 99.60% |
| ASV70:Staphylococcus | [Staphylococcus caprae strain DSM 20608 16S ribosomal RNA, partial sequence](https://blast.ncbi.nlm.nih.gov/Blast.cgi#alnHdr_645322521) | 100% |
| ASV75:Staphylococcus | [Staphylococcus hominis strain DM 122 16S ribosomal RNA, partial sequence](https://blast.ncbi.nlm.nih.gov/Blast.cgi#alnHdr_310975092) | 99.60% |
| ASV108:Staphylococcus | [Staphylococcus haemolyticus strain JCM 2416 16S ribosomal RNA, partial sequence](https://blast.ncbi.nlm.nih.gov/Blast.cgi#alnHdr_631252147) | 99.80% |
|  | [Staphylococcus haemolyticus strain SM 131 16S ribosomal RNA, partial sequence](https://blast.ncbi.nlm.nih.gov/Blast.cgi#alnHdr_310975091) | 99.80% |
| ASV133:Staphylococcus | [Staphylococcus nepalensis strain CW1 16S ribosomal RNA, partial sequence](https://blast.ncbi.nlm.nih.gov/Blast.cgi#alnHdr_265678691) | 99.79% |
| ASV173:Staphylococcus nephalensis | [Staphylococcus nepalensis strain CW1 16S ribosomal RNA, partial sequence](https://blast.ncbi.nlm.nih.gov/Blast.cgi#alnHdr_265678691) | 99.79% |
| ASV207:Staphylococcus | [Staphylococcus nepalensis strain CW1 16S ribosomal RNA, partial sequence](https://blast.ncbi.nlm.nih.gov/Blast.cgi#alnHdr_265678691) | 99.58% |
| ASV211:Staphylococcus | [Staphylococcus capitis strain JCM 2420 16S ribosomal RNA, partial sequence](https://blast.ncbi.nlm.nih.gov/Blast.cgi#alnHdr_631252150) | 99.80% |
| ASV409:Staphylococcus | [Staphylococcus edaphicus strain CCM 8730 16S ribosomal RNA, partial sequence](https://blast.ncbi.nlm.nih.gov/Blast.cgi#alnHdr_1397641709) | 99.80% |
|  | Staphylococcus saprophyticus subsp. saprophyticus ATCC 15305 = NCTC 7292 16S ribosomal RNA, partial sequence | 99.80% |
| ASV617:Staphylococcus | [Staphylococcus ureilyticus strain CK27 16S ribosomal RNA, partial sequence](https://blast.ncbi.nlm.nih.gov/Blast.cgi#alnHdr_310975182) | 99.80% |

BLAST searches were conducted on 26 March 2026 to identify the closest species-level matches for selected amplicon sequence variants (ASVs) based on 16S rRNA gene sequences. The table lists the top matches with the highest percentage identity per ASV. Only matches with 100% identity or the highest available match (if <100%) are reported.
